# Dr. Gardette and His Proposition Again

**Published:** 1852-07

**Authors:** 


### EDITORIAL DEPARTMENT.

***Dr. Gardette and his Proposition Again.***—Since our last issue, Dr. Gardette has published in the Medical Examiner, a long and angry defence of his conduct and views with regard to dental education.

From the first we have been exceedingly reluctant to enter into controversy with that gentleman, for it was evident that his opinions were held as a permanent part of himself, and would be defended with all the ardor of a vehement nature, rather than with the fairness of a calm and dispassionate mind.

That he would confound criticism with resentment, and abuse argument with personal assault, was nothing more than we expected, and that in our attempts to defend dentistry against a most dangerous scheme of amalgamation with ordinary medicine, to which it was to surrender whatever of respectability it had acquired in years of struggle, and from which it was to receive nothing in return, we would bring upon ourselves present abuse and permanent enmity was what all experience taught us to anticipate. Nevertheless, we confess we were not prepared for such an ebullition of spleen as the recent publication in the Examiner, and for the sake of our former sincere regard for Dr. Gardette, we profoundly regret that he has permitted himself to make such an exposure of his feelings and temper. Of him we have always spoken considerately and respectfully. We appeal to our readers whether we have written a sentence, in all the controversy, at which a reasonable opponent could have taken offence? If any such can be shown, we will gladly retract it; but we are not conscious of any such utterance as calls for apology. Of us Dr. Gardette has written in terms the most offensive that a respectable journal could be expected to publish. That he has rather insinuated than asserted his meaning, neither lessens the offence nor conceals the purpose. We see in this, nothing more than the effect of the necessity that is laid upon contributors to reputable journals to avoid coarseness in the manner of their invectives.

These insinuations we cannot notice more particularly without humiliation, and we therefore will leave them, without attempt at defence, to find a lodgement in any mind sufficiently unoccupied and greedy of evil to give them reception. It is sufficient for an honest mind, "*Nil conscire sibi, nullâ pallescere culpâ.*"

There is one statement made by Dr. Gardette, in which the readers of the Journal may feel somewhat interested. He charges us with refusing to publish a scientific article because it contained doctrines adverse to our own. In reply to this very grave accusation, we can only say, the Doctor has been misinformed. The article to which, we presume, he refers, was a review by an anonymous, though certainly, a very able writer, of a portion of the proceedings of the Mississippi Valley Association of Dental Surgeons, relating to questions propounded by Dr. Drake, concerning the cause of dental caries. It was rejected for a different reason, altogether, than the one stated by Dr. Gardette. Indeed, that had nothing to do with our refusal to publish it.

Our opposition to Dr. Gardette's scheme is based upon the fact, that it does not, and cannot provide for that thorough practical training so indispensable to a student of dentistry. If, then, it does not do this, the institution of a chair of dental surgery in the different medical colleges of the country, *ostensibly* for the purpose of fitting men for the practice of dentistry, would, as a necessary consequence, *lower* rather than *elevate* the standard of professional qualification. We are far from underrating the value of medical knowledge to the dentist. We were among the first in this country to advocate it, and have written, perhaps, more than a hundred pages upon the subject, which, we are happy to believe, have been instrumental, in some degree, in awakening the attention of the profession to its importance. With a view of elevating the standard of qualification, provision was made in the constitution of the Baltimore College of Dental Surgery, for teaching *anatomy*, *physiology*, *pathology*, *therapeutics*, and the general principles of *surgery*—thus extending the curriculum of dental education far beyond what was before generally deemed necessary for the successful practice of this specialty.

Now, we claim, that the course of instruction which we advocate, is much *broader* and more *complete*, than that contended for by Dr. Gardette. If it could be done, we would require every student of dental surgery, to graduate both in medicine and dentistry. But we know this is impracticable, as it would be requiring twice as much of the dentist, as of the physician. We would also extend the curriculum of medical education, and require a full and thorough course of instruction in dental *anatomy*, *physiology* and *pathology* to be given either by a separate professor, or append the duty to one of the other chairs. This knowledge would certainly be of importance to the general practitioner, and having it, he would be more ready to admit to equal consideration the claims of the educated dentist. But the idea of fitting men for the practice of dental surgery, in the manner as proposed by Dr. Gardette, is a chimera which can never be realised.

